# Physiognomy of the Insane
*Translated from the *Annales Medico-Psychologiques,* March, 1863.


**Published:** 1863-07

**Authors:** 

**Affiliations:** one of the Physicians to the Quatre Mares Asylum; Corresponding Member of the Medico-Psychological Society.


### Art. VI.—PHYSIOGNOMY OF THE INSANE.*

By Dr. Laurent, one of the Physicians to the Quatre Mares Asylum; Corresponding Member of the Medico-Psychological Society.

"The study of the Physiognomy of Lunacy is not the indulgence merely of idle curiosity; it assists in developing the character of those ideas and emotions that constitute the accession of that malady. What interesting results may not flow from such an investigation?"—*Esquirol.* Vol. ii., Chap. 12.

### I.

What part is moredeserving of the careful attention of the physician than the countenance? What study more important for him than that which has for its object the special expression of the several characteristics that are there met with?

In truth, who does not fix his eye and mind upon this centre of the several elements of which man is composed, or, as Aristotle expressed it, upon this miniature of the entire human being? Lavater, who made physiognomy the special object of his research, discovered in the countenance a microcosm, accurately representing the king of creation. According to him, in the face may be seen all the signs of the power of the understanding, the indications of its moral force, together with its desires, its irritability, the sympathies and antipathies of which it is susceptible, the power it has of attracting or repelling external objects; in fact, the condition of its physical and animal forces. No one has pursued the study of physiognomy more than the Zurich moralist; and it is to be regretted, as Moreau de la Sarthe justly said, in his fine introduction to his works, that Lavater had not the anatomical knowledge of a physician.

---

* Translated from the *Annales Medico-Psychologiques,* March, 1863.

The importance of physiognomy certainly did not escape the observing genius of the ancients, for the study of the indications furnished by facial expression occupies a considerable space in their writings. Hippocrates, in his work on *Prognostics,* takes great pains to describe all that relates to the face, and so likewise have his commentators and the most illustrious subsequent representatives of the medical profession.

Notwithstanding, it will readily be conceived that from the new impulse latterly given to the exact sciences, materials would be added quite enough to form a compendium of more solid and less problematical knowledge. Such has been the fact; and without citing the names of all those who have transmitted to us their observations on this subject, I shall mention Quelmatz, who, in 1748, sustained at Leipsig a thesis, entitled *De prosoposcopia medica*; François Cabuchet, who published in the year X. a series of representations of the various expressions of which the face is capable; Landré Beauvais, and Double, who have left us some detailed essays on the subject of semiology. Sir Charles Bell has brought forward the greater part of the ideas and descriptions made known in the works of Lavater by Moreau in his essay on the *Anatomy and Philosophy of Expression.* All treatises on general pathology mention the importance which Jadelot, the famous physician to the hospital for children, attached to the various expressions or salient lines in the face, as well as the significance he gave to them in forming his prognosis. Kælp, in a work entitled *De facie in morbis,* carried still further the study of these signs, and established numerous subdivisions. Lastly, Dr. Duchenne, of Boulogne, employed the electric current in the study of the facial muscles, and thus endeavoured to establish the real functions of this system.

If physiognomy be an interesting subject for the study of the physician, it is more especially in that branch of the art which owes its progress to the talents of Pinel and Esquirol. In the investigation of mental alienation it is especially important to consider the numberless and frequent modifications manifested in the countenances of individuals under the diverse impressions and varied emotions experienced. Here the subject becomes much more intricate. The physical condition suffices no longer. We must not confine ourselves to the emaciation of the countenance, the greater or less extent of colour in the cheeks, the degree of tone in the muscular fibres. We must connect these different modifications with those of the mental condition. We have, consequently, to examine the harmony existing between each constituent part of the visage, and its relative significance with the manifestations of an active, intelligent, and sensitive being. We must appeal to the analysis of each of the mental faculties.

I do not think any one will doubt that there can be a more useful study for the mental physician. Long ago physiologists demonstrated that the face is a mirror of the health of both body and mind; and the science of the relation of the physical to the moral has taught us that the actions of the mind, although so numerous, are represented in all their gradations by the physiognomy, and that their manner of reproduction is subject to invariable laws. It is on this account that the passions have the same expression throughout the entire human race. If disturbances so profound and abrupt as the passions are apparent to the generality of mankind, if joy and grief act constantly through the same nerves, and on the same muscles, it follows that the other acts of the soul have recourse, for their demonstration, to similar instruments. If these manifestations are not always apparent it is from want of ability in the observer; and the physiognomist ought to be gifted with extreme delicacy of perception, because he has to do with phenomena, it matters not whether normal or morbid, which very readily escape detection. We are thus obliged to confess that we meet in the physiognomy with the same discord that is noticed in a more elevated sphere, and are reminded that the various degrees of delirium and insanity remain perceptible on the countenance.

The absurd pretension is not, however, advanced, that it is possible to read in the countenance all that an individual can think about, or every psychical fancy that may arise. Ideas and combinations of ideas, or acquired knowledge, never will be recognisable in the countenance. But the degree of sensibility, the different moods of that faculty, the capacity of intelligence, the greater or less developed influence of the will—these are subjects upon which, by means of prosoposcopy, the observation of man may throw light both in the normal and the pathological condition.

Shall I be progressing too fast if I endeavour to show that the mental trouble of the lunatic is depicted on his countenance? The following proof may be permitted. Ask an intelligent and clever artist to portray the features of a lunatic whom he has known previous to the invasion of his malady; to depict him such as he was before his mental faculties were injured. Follow him while he traces the outline during the analysis of the countenance that he is obliged to make in order to reconstitute a being endowed with reason and free will. What a beautiful study of the relations between physiognomy and the mental faculties! Unfortunately, among the medical men who devote themselves to the speciality, there are very few artists who have the power of depicting the physiognomy of the patients under their care, and consequently of comparing the expression manifested at different epochs. Many characteristics are forgotten; one remembers only the most prominent, and the pen is not capable of describing all the important peculiarities. The artist's pencil is essential. The possibility of supporting my proposition may be conceived by employing the method I have indicated.

The celebrated Guislain, who himself took several portraits of lunatics, which were afterwards engraved in his work (*Leçons orales sur les phrénopathies*), would not have insisted in so marked a manner on that which he styles the *mask* of the madman, if his acquaintance with art had not allowed him to analyze the changes of the countenance. It must be confessed, notwithstanding, that his physiognomical studies are still imperfect. He was not the only specialist who saw the importance to physiognomy of the art of design. Esquirol realized the thought by adding some portraits of insane persons to his immortal work. He attached great importance to such pursuits. The illustrious practitioner intended to publish more detailed observations on this subject, and for that purpose had the portraits of more than two hundred of the alienated taken. Dr. Morel, in his *Clinical Observations*, has also inserted a great number of sketches. In each of these representations, it is to be regretted that, after having pointed out at which epoch of the complaint the artist was employed, the same individual was not depicted during the several stages of the mental disorder, as well as when convalescent. Esquirol did this in one case of mania only.

This gap in psychopathical symptomology is being daily filled up. I saw, in September, 1860, with the greatest satisfaction, at the Stephensfeld asylum, a room where Dr. Dagonet employs himself in taking what appear to him the most striking types of disease. M. Morel, has just constructed, at St. Yon, a photographic studio, wherein he takes the various physiognomies presented by the alienated to the eye of the physician.

In 1858, the late Dr. Ferrus, Inspector-general, caused a daguerreotype to be taken of the face of a madman who killed the much respected Dr. Geoffroy, then the chief physician. Three portraits were taken in different positions, one full face, another three-quarter, the third in profile.

I should very much have liked to append to this essay a certain number of photographs, and thus complete, by such satisfactory proofs, the researches to which for many years I have applied myself, for pictures are more easy of comprehension than all the verbal descriptions at one's command. I must produce somewhat later this indispensable complement to the present work.

II.

In medical phraseology the word *physiognomy* (φύσις, nature; νόμος, disposition, law) expresses nature's manner of being, the natural facial expression of the individual, both physical and moral; the expression of the entirety, of the combination of the special attribute of each constituent portion of the face, of the peculiar expression of every modification, whether transient or fixed, of these same parts.

But in psychopathical prosoposcopy we must understand, under the name of *face*, something more than is ordinarily understood by this term of topographic anatomy, or, at all events, we must add, in the study of the physiognomy of the insane, an examination of the different parts which have an important though distant relation with the countenance—such as the head in general, the hair, ears, &c. Each of these organs contributes more or less to complete the expression of the physiognomy, which besides, whatever people may think, very imperfectly exists, if we try to separate them.

The face, strictly speaking, is made up of the forehead, the eyes, the nose, cheeks, mouth, and chin. It is founded on an osseous framework, which is not without its value to the observer. Upon the skeleton are fixed the numerous muscular fibres, the movements of which express the varied changes of the mind. No other part of the human body has so great a number. There is no part richer in nervous filaments, and consequently of which the sensibility is more developed. In fact, without speaking of the nerves of special sense, we meet on the one hand the trifacial nerve, which gives sensation by means of its ophthalmic and superior maxillary branches, and to some extent by its mixed offshoot; and on the other, the three motores oculorum, the masticator (the nonganglionic portion of the inferior maxillary), and the facial, which preside over motion. Many other parts, less important to our present consideration, and interspersed among the preceding, are finally covered, as well as those I have just summarily pointed out, by that protecting and sensitive covering which we call the skin.

That which is styled in the language of physiognomy the *visage* is only one portion of the face, and extends from the upper lip to the top of the forehead.

The pursuit of physiognomy would be indeed a vain pretension if we left unnoticed the numerous pathological changes to which these parts are subjected. In fact, the morbid alterations that may happen should be kept constantly before our eyes. This is the most serious objection that has ever been advanced against the exactness of this study. I shall do no more than reply, that by analysis we are able to distinguish the cause of a pathological change, and that we may, by reason and attention, re-establish the primitive symmetry that has been destroyed, and reconstruct the countenance in its natural condition. Therein especially consists the talent of the observer, who divines the general expression from the physiognomical data thus acquired.

But the principles essential to psychopathical prosoposcopy are not limited to normal and pathologic anatomy. The alienist, more than any other physician, ought to possess an amount of philosophical knowledge sufficient to enable him to apply to the physiognomical expression of every period of life the instinctive tendencies as well as voluntary and intellectual manifestations or operations that belong to it. It is only on such conditions that it is possible to treat a mental affection in the proper way, if we do not wish chance to determine the results obtained and the experience deduced.

Every age is subject to invariable physiological laws. Their application is not attended with difficulty, except under the influence of a certain number of very variable causes, the study of which forms the subject of public and private hygiene. Moreover, these causes must be extremely powerful, in order so decidedly to influence the established order of nature. Psychopathical prosoposcopy is a verification of this admirable code.

Let us follow for an instant the parallel of the development of the physiognomy and of the intellectual being. This will be to us a paragraph preliminary to a description of the morbid manifestations we have presently to examine.

Man on the threshold of existence appears up to a certain point in the condition of an individual, who, about to visit a stranger whom he has never seen, and whose virtues and failings have never been described to him, is at the time of entrance in a high state of incertitude as to the manner he should assume on presentation, and the reception he is likely to meet with. The physiognomy of an infant at birth has clearly an expression of general doubt about the powers that preside over its material and psychical existence. We find no indication of the disposition afterwards manifested. It is an entirety containing in fact the elements of a differential nature that will be developed eventually, according as the various circumstances are favourable or not. The senses have as yet received no impression: the perceptions are confused. Instinctive life alone is powerful; sensibility is rudimentary. The countenance is incapable of receiving the impress of the sensations of hunger, of thirst, of pleasure, or grief. The forehead has neither the expansion nor contraction which will show themselves hereafter as the consequence of agreeable or disagreeable sensations. The eyelids, scarcely opened, display immovable pupils quite unfit to measure the amount of luminous rays adapted to the retina. The mouth has only the conservative movement of suction, and the repose consecutive to it. The lineaments of the face have yet to be engraved. The intellectual and voluntary are nearly absent.

But let a few weeks or months elapse, and see how complete a revolution has been effected. The various emotions make their way, and bring us the materials for solving this complex problem. We begin to discern which part appears set apart for natural instinctive or intellectual tendencies, if they have not been swerved from the purpose by pernicious influences. We already notice an aptitude for acquiring. By degrees the head takes a special shape; the osseous portions solidify more and more; the face becomes more or less elongated or round, and acquires a special type; the hair assumes its definitive colour and mode of insertion. The osseous protuberances attain their prominence; the fleshy masses are exercised in movement, and become proportionally developed. The repetition of these mechanical acts with their consequent traces, enables us to analyse to which faculty they are due. The expression of the look becomes the complement to these manifestations.

In proportion to the advance of age, the signs become less mobile or fugitive, and more easy of comprehension. Up to between twelve and fifteen years there comes to be painted on our canvas, in the portion reserved for sensibility, a greater or less lively impressionability, a constant agitation resulting from irresistible impulses of the instinct—a curiosity proportioned to the ignorance and the intellectual activity of the child, an imitative faculty in ratio with the power of psychical force. We there perceive the development of many sentiments and passions, such as sympathy and antipathy, the source of benevolent and malevolent affections; hatred, hope, fear, emulation, envy, timidity, bashfulness, pride, and obstinacy. The progression of intelligence is marked by distinction of sensations, birth of ideas, efforts of imagination, consolidation of the memory, the variable possibility of attention. The will, as yet, exercises but a feeble sway, and appears only at short intervals, at first by what is vulgarly called caprice, subsequently by the direction of the attention, the recal of past experience, the operations o. determination.

But it is really only after the epoch of from twelve to fifteen that physiognomy becomes of importance to the observer. This arises from the simultaneous influence of the formation of both moral and intellectual habits, and the progressive development of the imagination. The character which the physiognomy has acquired at the termination of that period is preserved evermore. It experiences some modifications: but these, although they may change it a little, never destroy the type, which, so to speak, has arrived at its zenith at the end of adolescence. We have left the child eager after every kind of moral and physical progress. The sensibility becomes more refined by the advantages of education and social converse; and we can admire at that age, more than others, the importance of the part it is destined to play as a provocative power, as a medium between the external and internal worlds, as a monitor of the aims of our existence, and as a necessary condition to the struggle which human liberty must sustain. A new sentiment holds an elevated position and influences all the faculties; a sentiment which has relation to all beings of an opposite sex and of the same age—love—which may assume enormous proportions, and become a passion ruinous to the entire individual. We notice, also, all the degrees and diverse modifications of the sentient being. "The intellect does not yet arrive at its height of development, but it possesses all the kinds of faculties that it can have. The judgment is sufficiently developed to encounter the most arduous difficulties of human knowledge in science and art. The adolescent can learn everything, but he cannot yet discover and invent all that the human mind can originate. He cannot yet sufficiently observe and reason. The dominant intellectual faculties are memory, which is happy and faithful; imagination, which is lively and brilliant, although little controlled by judgment. Hereafter will come its turn of superiority, but that time has not yet arrived."*

Neither has the will attained the force reserved for maturer age. Insensibly the individual acquires the consciousness of power to make determinations; he essays to give direction to his intellectual acts; he searches, at the cost of long and painful efforts, to ratify his empire over the intellect. He already perceives the extent of the responsibility attached to the consequences of his resolutions and actions.

Let us once again consider with what precision the physiognomy obeys the orders of the soul! The affective sensations, (pleasures and pains of the body, pleasures and pains of the spirit, pleasures and pains of the heart), the passions, have each one there its special representative, and in that place take right of domicile, according as the natural or acquired disposition of the individual, and an infinity of causes that are too numerous to be

---

* Gerdy.

here enumerated, determine their greater or less frequent or constant reproduction. Their traces are proportioned to their duration. As constituent elements, we may mention the dilatation, the contraction, the tension of the features, the motion and repose of each of the mobile portions of the face, the vivacity, the languor of the look, the fixity or variety of colour. It is at this age that the characteristic sign of the intelligence is best reflected, and this image will be more or less perfect according as the psychical influence triumphs over the animal nature, that the human being feels more or less deeply the influence of an upper world, and as his heart sympathises with that which is noble, beautiful, and generous.

To the organs that belong more especially to animal life, is due the expression of gross and bad thoughts, of wild and cruel passions, of which the lower part of the face is the most faithful mirror. The more noble sentiments seem to converge towards a higher region, and aid essentially towards the personification of the beau ideal, which is the most perfect character of that period of life. The intellectual and voluntary forces are specially represented within a triangle having the two eyes for a base, and the summit in the centre of the forehead; but the intellect is above all interpreted by the eyes.

During the two following periods, wherein youth and manhood are united, the intellect and will attain all their power. Imagination, which we have seen so influential in adolescence, so ready to combine ideas, submits to reason. In the second period, inspiration takes a secondary place with most men, and is omnipotent only with some individuals. Moreover, this creative faculty rarely persists in a high state of development, without detriment to such important faculties as the judgment and moral sensibility. Reason is really the sovereign that ought to reign supreme in the psychological domain of intellect; it has at its command the intellectual forces of attention, abstraction, comparison, generalization, induction, and deduction. The will is enthroned close beside him, and the two united become the source of the dignity of man. Meantime, although shorn of its beams, instinct is never entirely lost, and as Collineau has well expressed it, " man, with his tastes and desires so numerous and diverse, has never sufficient instinct to be independent of intellect, nor enough intellect to need instinct no longer."

But parallel with the development of the moral conscience, will be found to run that of a great number of passions that properly belong to this page of the physiological history of man, such as cupidity, ambition, debauch, love of gaming, and the horrible emotions it brings in its train; hatred, jealousy, and with them dissimulation, hypocrisy, proofs of the intimate knowledge that man possesses of his power to control his passions, and in the words of Rochefoucault, "the homage paid by life to the will."

Descuret has designed a chart of the qualities and defects that are principally met with in the chief professions, adding the best marked advantages and inconveniences which are presented by each of them. An examination of this precious document cannot fail to be useful to the medical philosopher in investigating the present subject.

This is the proper place to describe the mask pertaining to each passion, and every psychological type; but I thought I should be travelling out of the course I laid down for myself by detailing over and above it an essential part of physiognomy that may be studied in special works.

Let us proceed to the more advanced periods of human existence, the age of decline, senility. The age of decline merits a special description in woman. It is an epoch of profound perturbation, of an organic motion with variable tendencies, which may determine in her well-marked disturbances of the mental state, or recal to the healthy condition an economy the victim of sickly modifications dating from an epoch often long past. The countenance does not feel it less than the organism and the intellect. With man the revolution is less manifest, and may almost be passed over in silence. Although the aggregate material receives different impulses, the psychical element does not perceive, in a very distinct manner, the result of the work which has been effected until old age appears.

This latter has well-marked characters. It has been the subject of frequent meditation. Diverse philosophic theories have been founded on it. It is the most positive proof we can have of the highly natural division of the three psychical faculties, a division the French school has especially employed itself in developing. It is our irrefragable proof of the dynamic alliance of the two diverse principles of nature, the material whole and the immaterial being. Besides, in considering the more lofty faculties, intellect and will, we see that both judgment and reason persist the longest and ordinarily preserve their superiority over the other faculties. We have seen the imagination decrease: that decrease continues. The exclusive products of imagination are only met with exceptionally, and the works of the learned are the result of experience and reason. Memory is also enfeebled; the organic element, which evidently plays an important part in these intellectual phenomena, loses more and more of its power of preserving recent impressions. The recollection of distant facts remains a long time, and the association of ideas is attached not less to anterior acts. The will also remains well developed, when the individual has not given up in the course of preceding years the reins of his free will to the despotism and disturbing influence of passion. Sensibility has special manifestations. Instinct has been superseded by habit; the affections are limited; the powerful lever of love is nearly annihilated, and if we do not meet with temperance, moderation, and wisdom, we find in their place egotism, avarice, envy, and misanthropy.

In a physiognomic sense, we notice very remarkable changes in the countenance; wrinkles come to furrow it in all directions, and the furrows partake of the impulse which has presided over the moveable parts. We may notice these numerous pathological changes. The falling in of the jaws gives a new aspect to the face, which nevertheless loses naught of that which existed previously.

The deterioration of organs, material slaves of the psychical element, becomes an insurmountable obstacle to the manifestation of that power. The obliteration of the senses takes off and destroys the impressions produced, and the outward world no longer determines the provocative excitation of the activity of intellect. On the other hand, the natural messengers of the orders given by the latter become incapable of carrying out the functions which pertain to them; hence this new condition appears more or less early. It may even happen that man is reduced to the lowest condition, and degraded below the brutes. The facile expression comes into relation with this intellectual decadence, and the countenance of the man thus debased exhibits merely an assemblage of features inert, faded, and without other signification than obliteration. This last termination, let us hasten to declare, for the honour of humanity, is not the rule; most frequently this decay is limited, and, in the midst of the ruins of past glory, both intellectual expressions and ineffaceable affections may yet be discovered.

Such is a rapidly executed sketch of the relations between physiognomy and the psychical development. The progress of these phenomena does not always follow the laws of progression we have just pointed out, but is often stopped at one period or another. Hence the great difference observable in the intellect of various individuals. Whatever it be, the facial expression is always in accordance with the intellectual element.

The study of physiognomy enables us to distinguish both general and individual types. Among the former we recognise, in the expression of each passion, &c., those that are confined to certain races, nations, or families. The latter apply properly to each individual man. It has been said that no two men resemble each other, and hence some have felt justified in asserting the utter fallacy of physiognomy. Every physiognomy forms an harmonious whole resulting from very diverse influences. We can distinguish, by observation, beyond this complex assemblage, the co-ordination of general types, and in accordance with the relations and development of physiognomical character which belong to them, establish the predominance of one type over the rest. By this means reason will enable us to discover the individual as he really exists.

It is not so easy as people imagine to confound a simulated with a real expression; and civilization, which imposes on a man who would live in society a nearly constant dissimulation of his mode of viewing and feeling, is powerless in hiding the real state of the mind, and lets the penetrating eye perceive its pantomimic artificiality. Every sentiment has a special mask that cannot be perfectly imitated. For this reason a searching examination will always enable us to perceive a characteristic unsteadiness in the movements or acts that denote the sentiment, an exaggeration of the expression, or the absence of one or many of the essential features of its reproduction. The effrontery of the hypocrite arises from his belief that his chicanery cannot be detected, and, in fact, he succeeds in his aim with one who has only very superficial notions of physiognomy.

After these observations I hasten to the physiognomy of the alienated, and to reply to the question which doubtless has been often put to me from the very commencement of my work—Has the madman a peculiar physiognomic type, and can it generally be recognised? I answer: Yes.

Lavater, whom I cannot avoid invoking on such a subject, proposes the following experiment. Three different portraits are to be taken. The face in each is to be divided into three horizontal portions: the first to contain the forehead, the second the nose, the third and lowest portion from the nose to the chin. The next operation is to adjust the nasal portion of the second to the frontal part of the first portrait, as well as the inferior section of the third. By this arrangement we infallibly obtain the physiognomy of an insane person. Hence he concluded that there was a manifest defect of harmony in the countenance of the alienated. This proposition is perfectly true, and the proof thus furnished by the illustrious physiognomist is, in our mind, the foundation of that which I shall endeavour to explain in this work. But we cannot admit the consequences that he thought might be drawn from his theory of beauty. It is not a theory we invoke in our favour, but facts which serve and will serve to establish what we advance. Thus the development of the frontal region of the nose or chin cannot be adopted, we believe, to indicate insanity as Lavater proposes. We seek another criterion, and believe we are able to find it in the *tout ensemble* of the physiognomy, in the more or less evident discord in the movements of the face. However, we may say that we have met with this disproportion of countenance very frequently among individuals with some peculiarity of character or actions ; and that this disproportion is met with among those who in the world are designated eccentrics or originals. But we cannot affirm that it is here an indication of insanity.

Among the alienated the face is often well-proportioned, and betwixt eccentricity and madness there is a great gap.

If we recal to mind what has just been said respecting the type belonging to each individual, and the special physiognomy formed in accordance with the development of his intellect and moral habits, it will be easy to understand what is here advanced. The relations of a patient point out clearly to the physician that the invalid has something unusual in his countenance, something they cannot precisely define, but which appears to them extraordinary. It is on this account that Dr. Damerow, in an article on mimicry and physiognomy, published in the *Allgemeine Zeitschrift für Psychiatrie*, 1860, advises a consultation with the family on the return of the physiognomy to its ordinary condition. This is good counsel, and should be adopted. The physician only sees the person confided to his care when the disease has greatly modified, and often for some considerable time, the normal type. Besides, the shades vary so much, that it becomes in certain cases extremely difficult to recall the physiognomy of healthy individuals, notwithstanding the knowledge of character which serves to assist in their elucidation. As proof of the general physiognomy of the alienated, I may mention, *en passant*, the sentiments of ordinary people who visit our asylums, and who discover something incongruous in the patients' countenances. Women especially, by reason of their greater sensibility, point out to us very remarkable shadowings.

I will now endeavour to describe in what this want of harmony depends, by reference to the parts of the face that appear to me most significant.

The late Dr. Geoffroy, who has unfortunately published none of his observations on insanity, has oftentimes called my attention to the importance of an examination of the countenance of the alienated. The eye, in truth, is the most expressive organ of the face, as well as its most active feature. It can confront that which is agreeable, fly that which is displeasing, move itself in a variety of ways, exclude itself, by the closure of the lids, from visible nature. Frankness and dissimulation can be thus equally recognised. The criminal, notwithstanding all his audacity, rarely looks you in the face. His look is furtive, unsteady, sullen, and has an air of indecision that betrays him continually. The honest man has, on the contrary, an open countenance which produces confidence and engenders goodwill in those who see it. "Timidity casts down the eyes with a certain grace easy of recognition, goodness inclines them towards the earth, pride carries them towards heaven; they are set on fire and made menacing by anger; hope gently directs their gaze upwards; love makes them more brilliant, shades them a little, and throws them forwards." (*Belouino, des Passions*, p. 83.) Among the Chinese the judges place much reliance on the looks of the accused.

From these few words it may be perceived what importance the organ of vision has in the countenance, and what superiority of activity it possesses over the other organs of the senses. It also forms the centre of an arrangement of great importance. A certain number of parts surround and converge towards it. According to Charles Lebrun, chief painter to Louis XIV., the eyebrow is the portion of the face where the passions may be best recognised—it indicates the nature of the agitation observable in the eyes.

The mouth is another centre of action of the mobile parts. Without according to that organ so much importance as is assigned by Dr. Dorigny (*De la bouche humaine*), I am of the same opinion as Descuret and the principal physiognomists, that, after the eyes, the mouth is the most expressive of all parts of the face. We there find modifications of great value, corresponding to the passions of joy and grief, a state of mobility and of repose, a form which varies with certain situations, impressions, &c. The emotions of the soul that it is destined to portray are of no less elevated rank. Its office relates especially to the passions and appetites. It completes the signs conveyed to us by the eye and ocular apparatus. In the blind it acquires much more perfect expression.

The distinction between these two centres of action is very evident in what may be called the "forced" conditions of the mind. Falsehood and dissimulation may be distinctly recognised by the want of harmony of action between the ocular and buccal system, by the extraordinary mobility of the lips and their muscles. All the defects of physiognomic congruity relate more or less to these two centres. It is very easy to convince oneself of the importance to be attached to the co-ordination of expression furnished by the ocular and buccal centres of action. Consult those actors who have made a special study of facial mimicry. It is like taking a series of observations of oneself in a mirror, or on a sheet of ice, to carefully study our great dramatists on the stage. This want of harmony is explained by the impotence of a man who wishes to fix his attention upon several objects at once, and to direct in an harmonious manner all the motions essential to the expression of a sentiment which he really does not feel.

The investigation I have made on this subject among the alienated permits me thus to class under two fundamental heads the study of the modifications of the countenance. According to my own observation, the type of the lunatic in general depends upon the want of harmony between the expression of the centres of ocular and buccal action. But this want of harmony resembles that observed in dissimulation, although it is more complex. Besides, the duration of its manifestations is greater, and the circumstances are very different.

In the course of his malady the lunatic has many moments wherein the facial expression assumes the normal state, or nearly so. The reproduction of the normal and morbid conditions differs in accordance with the period of the malady, and other very numerous causes. There is no case of mental alienation wherein the want of symmetry I have pointed out cannot be demonstrated.

Besides the symptoms of defective harmony, there are others which are due to the influence of the organism, and which serve to distinguish the special types of insanity, as well as the period to which they belong.

III.

It has previously been shown that the physiognomy of the lunatic has a special character. In this section I propose to study the symptoms that can be presented by the face in each of its principal constituent parts.

I am inclined to allow that deformities of the head indicate an intellectual defect, or at least irregularity. The works of MM. Foville, Lunier, Gosse, Morel, Baillarger, &c., and those of a great number of anthropologists, as well as the researches which I have myself made on this subject, afford sufficient proof of what I advance.

1st. These deformities may be congenital, the sad effects of heritage, and allied to primitive intellectual debilities, as idiotcy, imbecility, and cretinism. We have met with diversities from regular microcephalus to macrocephalus and hydrocephalus, passing by all the groups which Dr. Gosse has so attentively studied.

2nd. Artificial deformities, resulting from injuries or erroneous practices which stop the free development of the intellect in a direct or indirect manner, and producing convulsive affections which almost necessarily induce mental trouble.

3rd. Lastly, subjective acquired deformities, proceeding from a perversion of the natural dynamic law under the influence of pathological causes, from want of symmetry in the activity of the individual. This absence of symmetry, which is of common occurrence, is always accompanied by an irregularity of the mental faculties, a peculiarity of character, an originality, without necessarily producing mental alienation. In some exceptional individuals, a greater development of one cerebral hemisphere has been found united with very large psychical capacity. (Bichat, Napoleon I.)

But although deformity of the cranium generally indicates an anomaly of intellectual actions, it does not follow that insanity is always associated with an ill-shapen skull. To maintain this would be a grave error. Many lunatics have the cranium well-formed and perfectly symmetrical.

Important elements are furnished to symptomatologists by the hairy system. Although asserted by Esquirol, the colour of the hair and beard has not appeared to us allied to one kind of insanity more than another. The popular saying that the head of the idiot never becomes grey, appears to us undeserving of confidence. But it is the condition of these products of secretion that should be considered. The softness or roughness of the hair and beard, their brittleness, dryness, or humidity, their smoothness or erection, their entanglement, agglutination, and length, their more or less complete change of colour, their neat or dirty condition, always accompanying special periods of the malady, should not escape the eye of the observer. The scarcity or abundance, the mode of distribution, the premature appearance,* more or less loss of these protecting organs, have a not less intimate relation with phrenopathic phenomena, and are very often allied with a primitive alteration (idiotcy, &c.).

The condition and colour of the skin have great value in the eyes of the alienist physician. I think it right expressly to insist on the symptoms furnished by this organ. I have noticed some very curious morbid phenomena. Professor Trousseau has specified in his clinical lectures some very important peculiarities in the functions of the skin manifesting themselves during head affections. After the example of this learned man, I must insist on this point. Colour furnishes signs well worthy attention. The skin of the face, and it is of this part alone I speak, may be dry and arid, the seat of herpetic scurvy and scaly eruptions, or may be moist with

---

* My worthy colleague and friend, Dr. Bulard, has noticed the appearance in women, at the epoch of commencing lunacy, of a larger or smaller number of bristles in the face, which have completely disappeared with the malady.

perspiration, or a liquid secretion of a more or less oily nature and of variable odour. Its colour is susceptible of numerous general or partial modifications. It may be pale. This pallor has divers shades, from pure white to the slightly yellow tinge (compared to that of straw or wax), or earthy, brown, and bronzed. It may be of every shade of red, from rosy to vermilion, violet and purple. But season, and exposure to the sun's rays, should always be taken into consideration.

The skin may have a greater or less tonicity, and the subcutaneous, subcellular tissue be more or less elastic. It also is marked by lines and furrows, which are of importance as indicating the amount of activity of the subjacent muscles. At first, during infancy and adolescence, few in number, their formation becomes fecund in proportion as age advances, which must be attributed to the thinning of the face or the loss of the mobile parts by age, sickness, passion, and deep emotion of the soul. I think it unnecessary to describe these furrows, which may assume different forms — horizontal, vertical, oblique, sinuous, and more or less close or parallel.

The organ of sight offers for consideration its form, movements, and expression. The eyes may be more or less prominent or depressed in the orbit; the aperture between the lids smaller or greater; the sclerotic, very apparent around the pupil, exhibits a variable bluish, yellowish, or red tinge; the dilatation of the vessels very evident. Little livid or black veins may be perceived on it. The conjunctival surface may be dry, humid, or moistened with tears; the pupils may be deformed by being equally or unequally dilated or contracted. Strabismus may be observed, a distortion of the eyes by which they look crosswise, either above, below, or to the side, twisting even during sleep. In the normal state the ocular globe is susceptible, under the influence of the will, of numberless motions in every sense, and these motions may have a longer or shorter duration; but in the morbid state, and without their owner's control, a sort of trembling, oscillation, or vacillation of the globe may be manifested, a kind of continual or permanent convulsion, in consequence of which, most frequently, little lateral, sometimes though rarely up and down, movements are given to the globe of the eye.

The expression of the eye calls for special attention. The eyes are sometimes lively and brilliant, sometimes sad and glazed. Often they have a soft, dreaming look, expressive of vacuity, uncertainty, or nonchalant calmness; at other times they become animated from the slightest cause, have a lightning glance, are haggard, insolent, full of audacity, fixed and inquisitive. Each of these expressions has a different intensity and duration, and responds to very different situations.

In accordance with the protrusion or sinking of the globe of the eye, the eyelids take shape—they are swollen or œdematous; have at times a very pallid colour, at others become red or blue; and exhibit wrinkles of diverse shape and in variable number. They may likewise be agitated by convulsion, or show a very significant immobility. Each lid may differ in the length and abundance of its lashes: the ciliary margin may be the seat of inflammation due to nervous excitation.

Occasionally the eyebrows are of fantastic shape. Sometimes little noticeable, sometimes strongly marked, they stand up on the forehead, or fall back on the eyes, curling after the style of moustaches.

The shape of the nose has also a pathological signification which should not be passed over in silence. Besides the colour and swelling or thinness of the fleshy parts of the proboscis, a careful examination should be made of the more or less easy dilatation of the nostrils, their mobility or fixedness, the tension or the retraction of their walls. Dr. Hofling* attaches much more importance to the signs furnished by the nose than to those given by the eye.

The mouth presents for examination the state of the lips, with their relative situation during repose, their volume, colour, dryness, or humidity. The motion of the mouth has a very important signification, and leads to a notable modification of the commissure of the lips. Permanent contractions, alternations of tension or relaxation, partial or general tremor, the diverse forms of spasm, deserve much attention. These manifestations have a very decided meaning.

What we have just said relative to the motion of the mouth and lips is applicable to all the locomotive system of the face. Tension or relaxation, continual or alternate movements, immobility, may appear in various grades in each of the facial muscles.

To facial symptomatology must be added also an examination of the parotid and auricular regions. We should carefully note the pallor, redness, and swelling of the cheeks; the colour, swelling, mobility, or immobility of the ears, as well as the appearance of sanguineous tumours of the auricle. Dr. Morel attaches much importance to the way in which the ears are fixed, and makes this one of the characteristic signs of his types of degeneracy.

---

* "Memoir on the Semiotic Indications furnished by the External Nose." (*Journal de Cooper*, 1834).

It is of some importance to let this physiognomical survey embrace the carriage of the head, which is often noticed to be variable, according as the individual has a more or less favourable opinion of his personality, and from numerous other causes.

Such are the different symptoms that the physiognomy of the lunatic presents to us. It is necessary to follow their manifestations in the numerous forms of mental alienation. We must remember that Guislain has forcibly insisted on the examination of the development and subsidence of the symptoms of mental disease. In a celebrated clinique he has classified the phenomena of morbid incubation, the phenomena of invasion, the phenomena of morbid progress, and stationary phenomena; phenomena of morbid decrease, phenomena of convalescence; and lastly, the phenomena announcing the transformation of the malady.

These several categories of morbid phenomena are noticed in each of the mental affections, and call for incessant attention on the part of the observer. It may be conceived that the symptoms we have above enumerated will vary in accordance with the period at which the patient comes under observation. Without ceasing to belong in a marked manner to a certain period, they will yet vary according to the dominant medical constitution—according to the medium from whence the affection arose, and a number of other causes which it is not necessary to mention.

---

